# Case Report: Fulminant aggressive NK-cell leukemia with hemophagocytic lymphohistiocytosis and multiple organ dysfunction successfully bridged to allogeneic hematopoietic stem cell transplantation

**DOI:** 10.3389/fimmu.2026.1994954

**Published:** 2026-10-28

**Authors:** Shutan Li, Chao Fang

**Affiliations:** 1 Department of Hematology, the Affiliated Hospital of Southwest Medical University, Luzhou, Sichuan, China; 2 Stem Cell Immunity and Regeneration Key Laboratory of Luzhou, Southwest Medical University, Luzhou, Sichuan, China; 3 Department of Hepatobiliary and Pancreatic Surgery, The Affiliated Hospital of Traditional Chinese Medicine of Southwest Medical University, Luzhou, Sichuan, China

**Keywords:** aggressive NK-cell leukemia, allogeneic hematopoietic stem cell transplantation, Epstein-Barr virus, hemophagocytic lymphohistiocytosis, JAK/STAT, P-Gemox

## Abstract

Aggressive natural killer-cell leukemia (ANKL) is an exceedingly rare and rapidly fatal malignancy of mature NK cells, frequently associated with Epstein-Barr virus (EBV), and its diagnosis is often complicated by hemophagocytic lymphohistiocytosis (HLH) that can obscure the underlying malignancy and delay therapy. We report the case of a 28-year-old male who presented with high-grade fever, pancytopenia, hepatosplenomegaly, and disseminated intravascular coagulation (DIC), with laboratory findings confirming HLH; bone marrow examination and flow cytometry identified an aberrant NK-cell population with a characteristic immunophenotype (CD2+, sCD3−, CD56+, CD16−), establishing the diagnosis of ANKL, and next-generation sequencing revealed mutations in JAK1, ASXL1, KMT2D, and FAS. The patient received sequential multimodal therapy, including a single administration of etoposide plus dexamethasone for initial HLH control, followed by two cycles of P-Gemox (pegaspargase, gemcitabine, oxaliplatin), and subsequently underwent successful haploidentical allogeneic hematopoietic stem cell transplantation (allo-HSCT) from his sibling, followed by one year of chidamide maintenance. Complete remission was achieved after P-Gemox induction and has been maintained with full donor chimerism at 35 months of follow-up. This case highlights that timely diagnosis and a sequential, multimodal treatment strategy can successfully bridge ANKL patients presenting with HLH to curative allo-HSCT. P-Gemox appeared to be well tolerated in our patient, and these findings underscore the critical importance of early recognition and prompt treatment initiation for improving outcomes in this devastating disease.

## Introduction

Aggressive natural killer-cell leukemia (ANKL) is a rare but devastating hematological malignancy characterized by the systemic proliferation of mature natural killer (NK) cells (1). ANKL is frequently associated with Epstein-Barr virus (EBV) infection and carries a dismal prognosis, with a median overall survival (OS) typically measured in weeks to months (2, 3). The disease is notoriously difficult to diagnose, not only due to its rarity but also because its clinical presentation is highly non-specific and often mimics severe infections or other hematologic conditions (4).

A particularly challenging aspect of ANKL is its frequent association with hemophagocytic lymphohistiocytosis (HLH), a hyperinflammatory syndrome driven by uncontrolled activation of macrophages (5). HLH can be the initial manifestation of ANKL in over 50% of cases, and its cytokine storm often dominates the clinical presentations, masking the underlying ANKL and leading to diagnostic delays and rapid clinical deterioration (6, 7). The prognosis for patients with ANKL complicated by HLH is even more grim than for those without (8).

Therapeutic advances have been made with the introduction of L-asparaginase-based regimens, which leverage the deficiency of asparagine synthetase in NK cells. The SMILE (dexamethasone, methotrexate, ifosfamide, L-asparaginase, etoposide) regimen has shown superior efficacy but is associated with significant toxicity (9). P-Gemox (pegaspargase, gemcitabine, oxaliplatin) has emerged as a less toxic alternative with comparable efficacy in NK/T-cell lymphomas (10). Allogeneic hematopoietic stem cell transplantation (allo-HSCT) remains the only potentially curative approach, with recent studies demonstrating improved survival in transplanted patients (11, 12).

Herein, we report a case of a young adult with ANKL presenting with severe HLH and multiorgan dysfunction who achieved complete remission with P-Gemox and successfully underwent haploidentical allo-HSCT. We also provide a comprehensive review of the literature focusing on molecular pathogenesis and therapeutic strategies. Although individual components of this management strategy have been described previously, this case is notable for the extreme severity of the initial presentation, with fulminant HLH and multiple organ dysfunction, and for the detailed longitudinal molecular and imaging follow-up, including serial PET-CT and post-transplant clearance of somatic variants. This case may therefore help inform the recognition and management of similarly affected patients.

## Case presentation

### Patient presentation and initial evaluation

A 28-year-old male presented with a 5-month history of skin petechiae and ecchymoses. He was initially treated with prednisone at a local hospital. Four months prior to admission, he developed persistent high-grade fever (peak 41 °C), accompanied by dizziness, fatigue, occasional blurred vision, chest tightness, and dry cough. At the local hospital, he was diagnosed with viral myocarditis, heart failure, pulmonary infection, anemia, thrombocytopenia, and hepatic dysfunction. Due to lack of response to treatment, he was transferred to our institution’s cardiology department. Given the suspected diagnosis of hemophagocytic lymphohistiocytosis (HLH), the patient was referred to the Department of Hematology for further management. The patient had no prior history of hematological malignancies, autoimmune diseases, or chronic infections, and no history of chemotherapy or radiotherapy. He denied tobacco and alcohol use and had no known occupational exposure to leukemogens. There was no family history of hematological malignancies or hereditary diseases.

On admission, vital signs were: temperature 39.8 °C, heart rate 132 bpm, respiratory rate 28/min, and blood pressure 88/51 mmHg. The patient was acutely ill with poor mental status and lethargy, and his Glasgow Coma Scale score was 13. Skin and scleral icterus were present, with scattered ecchymoses and petechiae on all extremities. A palpable left supraclavicular lymph node of approximately 1 cm was noted. The abdomen was distended with muscle guarding, tenderness, and rebound tenderness. The liver was palpable 1 cm below the right costal margin, and the spleen was palpable 3 cm below the left costal margin. Bilateral lower extremity edema was present.

### Laboratory findings

Laboratory evaluation at admission revealed severe pancytopenia: WBC 0.51 × 10^9^/L (reference range, 3.5–9.5×10^9^/L), NEU 0.45 × 10^9^/L (reference range, 1.8–6.3×10^9^/L), HB 61 g/L (reference range, 130–175 g/L), and PLT 1 × 10^9^/L (reference range, 125–350×10^9^/L). Biochemical tests showed significant hepatic dysfunction: AST 175.2 U/L (reference range, 0–40 U/L), ALB 21.6 g/L(reference range, 40–55 g/L), TBIL 114.8 μmol/L (reference range, 3.4–20.5 μmol/L), DBIL 71.9 μmol/L (reference range, 0–6.8 μmol/L), ALP 865.1 U/L (reference range, 45–125 U/L), and LDH 797.2 U/L (reference range, 120–250 U/L). Triglycerides were markedly elevated at 14.85 mmol/L (reference range, <1.7 mmol/L). Coagulation studies revealed prolonged PT (19 seconds; reference range, 11–14 seconds) and APTT (53.5 seconds; reference range, 28–45 seconds) with decreased fibrinogen (0.98 g/L; reference range, 2–4 g/L). Procalcitonin was elevated at 10.62 ng/ml (reference range, <0.05 ng/ml). EBV-DNA was positive at 1.89 × 10^5^ copies/mL (reference range, undetectable).

Cytokine profiling revealed markedly elevated inflammatory markers: IL-6 2586.02 pg/mL (reference range, <7 pg/mL), IL-10 2730.02 pg/mL (reference range, <5 pg/mL), and IFN-γ 478.85 pg/mL (reference range, <20 pg/mL). Soluble CD25 (sCD25) was 65,120 U/mL (reference range, <6400 U/mL), and NK cell activity was reduced to 3.1% (reference range, >15%) as measured by a flow cytometry-based NK cell cytotoxicity assay (CD107a degranulation). Ferritin exceeded 1000 ng/mL (reference range, 30–400 ng/mL). Troponin T was 0.015 μg/L (reference range, <0.014 μg/L), and BNP was 139 ng/ml (reference range, <100 ng/ml). Echocardiography showed left ventricular ejection fraction (LVEF) of 46%, 53%, and 62% at pre-treatment, post-chemotherapy, and pre-transplantation, respectively. Bone marrow smear demonstrated markedly hypercellular bone marrow with 19.5% unclassified cells. These cells were large with abundant cytoplasm containing vacuoles and granules, irregular nuclei, and coarse chromatin (**Figure 1A**). Hemophagocytosis was observed in the bone marrow aspirate, with approximately 1.5% of nucleated cells showing hemophagocytic activity, characterized by macrophages actively engulfing erythrocytes and platelets. Bone marrow Biopsy revealed hypercellular hematopoiesis (cellularity ~80%, fat ratio 4:1). Immunohistochemistry: CD3ϵ(+), CD56(partial+), TIA-1(+), Granzyme B(+), CD30(+), P53(mutant), Ki67(70%), CD4 (focal+ in a minor subset)), CD8(scattered+); CD5(−), CD20(−), CD7(−), CD123(−), CD100(−), CD38(−). EBER-ISH was positive (>80%), supporting ANKL. Flow cytometry identified an aberrant NK-cell population comprising 2.06% of nucleated cells, and the immunophenotype was consistent with ANKL: positive for CD2, cytoplasmic CD3 (cCD3), CD56, and negative for surface CD3 (sCD3), CD4, CD5, CD8, and CD57 (**Table 1**). The apparent discrepancy between focal CD4 positivity on immunohistochemistry and CD4 negativity on flow cytometry likely reflects differences in antibody clones, gating strategies, and the small size of the abnormal population; the flow cytometric result was considered definitive for the diagnostic immunophenotype.

**Figure 1:**
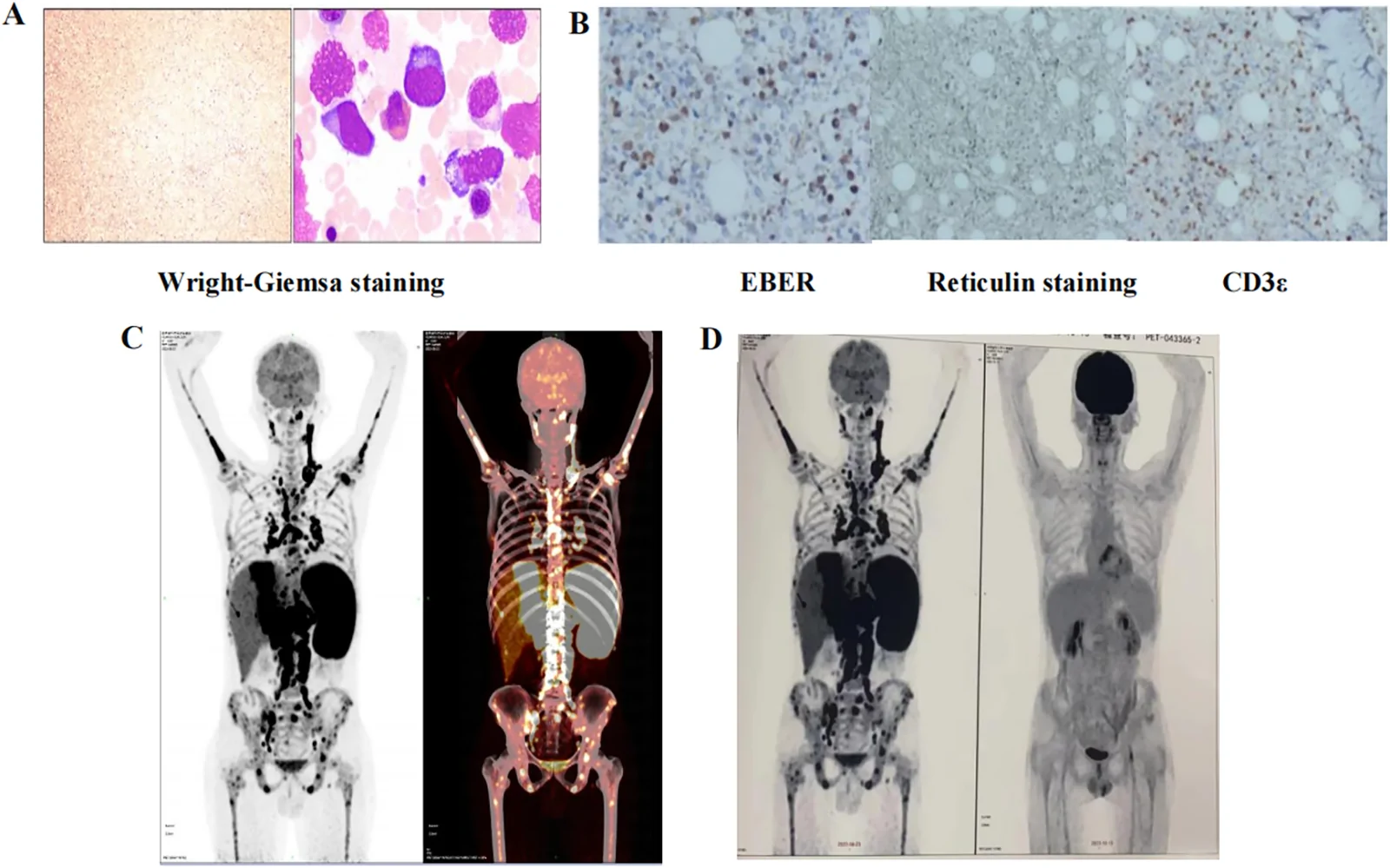
The bone marrow and PET-CT findings supported the diagnosis of ANKL. **(A)** Bone Marrow Aspirate. Markedly hypercellular; 19.5% unclassifiable cells with enlarged bodies, abundant cytoplasm containing vacuoles and granules, irregular eccentric nuclei, coarse chromatin, and rare nucleoli. **(B)** Bone Marrow Biopsy. Hypercellular (cellularity ~80%, fat ratio 4:1) with CD3ϵ^+^/CD56^+^/EBER^+^ lymphoid aggregates, supporting ANKL. Immunohistochemistry: CD3ϵ^+^, CD56(partial^+^), TIA-1^+^, Granzyme B^+^, CD30^+^, P53(mutant), Ki67(70%), CD4(focal^+^), CD8(scattered^+^); CD5^−^, CD20^−^, CD7^−^, CD123^−^, CD100^−^, CD38^−^. EBER-ISH >80%. **(C)** Pre-treatment PET-CT. Extensive lymphadenopathy with increased FDG uptake; hepatic lesions, splenomegaly, skeletal hypermetabolic foci, mesenteric/omental thickening, and left pterygoid involvement—all suggestive of lymphoma. Also noted: pulmonary micronodules, pleural effusion, pericardial effusion, ascites, and subcutaneous edema. **(D)** Post-2 cycles of P-Gemox PET-CT:.Significant reduction in lymphadenopathy and FDG uptake; spleen remained enlarged but metabolically inactive; mild skeletal hypermetabolism. Pulmonary micronodules with no uptake; minor fibrotic changes; post-catheter changes. Overall assessment: CR.

**Table 1:** Flow cytometric analysis of the bone marrow.

Parameter	Result
Total nucleated cells acquired	170,000 cells
Mature lymphocytes/nucleated cells	5.25%
CD3^+^ T cells/lymphocytes	89.59%
CD3^+^CD4^+^/CD3^+^CD8^+^ ratio	0.37
NK cells/lymphocytes	4.62%
CD3^+^CD57^+^ T cells/lymphocytes	20.71%
CD3^+^CD56^+^ cells/lymphocytes	26.04%
CD19^+^ B cells/lymphocytes	3.91%
KAPPA/LAMBDA	1.05
Abnormal cell population/nucleated cells	2.06%
Positive	CD2, CD56, CD38, cCD3
Negative	CD4, TCRγδ, CD5, sCD3, CD16, CD57, CD45, CD45RO, CD8, CD7, LAMBDA, CD23, CD19, CD200, KAPPA, CD20, CD10

Flow cytometric analysis of the bone marrow revealed an aberrant NK-cell population accounting for 2.06% of nucleated cells, characterized by large cell size and high side scatter (SSC), raising suspicion for ANKL.

Next-generation sequencing (NGS) revealed mutations in JAK1 (c.2108G>T, p.Ser703Ile, 11.3% variant allele frequency), ASXL1 (c.1934dup, p.Gly646TrpfsTer12, 10.1%), KMT2D (c.6040C>T, p.Gln2014Ter, 10.9%), and FAS (c.197-1G>A, 10.3%; c.355T>C, p.Cys119Arg, 10.4%). Chromosomal analysis showed a normal karyotype (46, XY).

PET-CT demonstrated extensive involvement: generalized lymphadenopathy with increased FDG uptake, hepatosplenomegaly with diffuse FDG uptake, multiple bone marrow lesions with increased FDG uptake, and left pterygoid soft tissue thickening with increased FDG uptake (**Figure 1C**). Pulmonary nodules, pleural effusion, and pericardial effusion were also noted.

A definitive diagnosis of ANKL with secondary HLH (13) was made.

### Management and transplantation

The patient was initially started on etoposide (VP16) and dexamethasone to control the hyperinflammatory syndrome. Empirical broad-spectrum antibacterial and antifungal therapy was initiated, together with supportive measures including transfusions, hydration, diuretics, hepatoprotective agents, electrolyte supplementation, and oxygen therapy.

Following confirmation of the diagnosis, the patient received P-Gemox chemotherapy (gemcitabine 1.6 g, oxaliplatin 160 mg, pegaspargase 3750 U). P-Gemox induction was well tolerated in this patient, with no clinically significant treatment-related toxicities observed. No dose reductions were required. The patient did not develop pancreatitis, thrombosis, or hypersensitivity reactions. A lumbar puncture was performed, with no evidence of central nervous system involvement; intrathecal chemotherapy (methotrexate 12.5 mg, cytarabine 50 mg, dexamethasone 5 mg) was administered prophylactically.

After two cycles of P-Gemox, the patient achieved complete remission. Response was assessed according to standard criteria for NK/T-cell neoplasms, incorporating clinical evaluation, bone marrow morphology, flow cytometry, EBER-ISH, and PET-CT. Complete remission was defined as the absence of morphologically detectable abnormal cells on bone marrow examination, negative flow cytometric residual disease assessment, EBER negativity on bone marrow biopsy, and complete metabolic response on PET-CT (Deauville score ≤ 3). The sensitivity of flow cytometry was approximately 10^−5^, and molecular monitoring was performed by NGS with a sensitivity of approximately 1–5% variant allele frequency. Accordingly, bone marrow examination showed no abnormal cells, flow cytometry was negative for residual disease, bone marrow biopsy showed no lymphoma cells with EBER negativity, and PET-CT demonstrated significant resolution of previous lesions with markedly decreased FDG uptake (**Figure 1D**).

The patient underwent haploidentical allo-HSCT from his 26-year-old brother (6/12 HLA-matched). Peripheral blood stem cells mobilized with recombinant human granulocyte colony-stimulating factor (rhG-CSF, filgrastim) were used as the graft source. Conditioning consisted of the FBA regimen (fludarabine 30 mg/m²/day on days −6 to −2, busulfan 3.2 mg/kg/day on days −5 to −4, and cytarabine 2 g/m²/day on days −6 to −3). The graft contained CD34+ cells 5.2 × 10^6^/kg and mononuclear cells 5.7 × 10^8^/kg.

The patient developed sepsis (Klebsiella pneumoniae) on day +11, which was successfully managed with antibiotics. Neutrophil engraftment occurred at day +20, and platelet engraftment at day +32. For GVHD prophylaxis, rabbit anti-thymocyte globulin (Thymoglobulin) was administered at a total dose of 5 mg/kg (2.5 mg/kg/day on days −3 and −2), together with cyclosporine, mycophenolate mofetil, and prednisone. All anti-rejection medications were completely discontinued after six months. The patient then received chidamide maintenance therapy for one year.

### Post-transplant follow-up

At the two-month post-transplant evaluation, blood counts and liver function had normalized. EBV DNA was negative, while CMV was detectable at 3.896×10³ copies/mL and was treated with ganciclovir. The patient was negative for JAK1, ASXL1, KMT2D, and FAS mutations. Chimerism analysis showed 99.66% donor cells. There was no evidence of acute GVHD. At six months post-transplantation, EBV became undetectable, and all anti-rejection medications were completely discontinued. Chidamide maintenance therapy was continued for one year and was completed without significant adverse events or dose reductions. No grade 3–4 adverse events, unexpected events, or treatment-related hospitalizations occurred during maintenance. The patient underwent regular follow-up with a total of six lumbar punctures and intrathecal chemotherapy administrations. At 35 months of follow-up after HSCT, the patient was doing well and remained in complete remission (CR). A detailed timeline of the clinical course, including key diagnostic and therapeutic milestones, is presented in **Table 2**.

**Table 2:** Timeline of the clinical course.

Phase	Time point	Event
Presentation & diagnosis	Onset	Skin petechiae and ecchymoses
	Local hospital	Misdiagnosed as viral myocarditis
	Referral to our institution	Diagnosis of HLH
	Bone marrow/flow cytometry/NGS	Diagnosis of ANKL
Initial treatment	VP16 + dexamethasone	Control of HLH
	P-Gemox cycles 1–2	Induction therapy
	Response evaluation	CR (PET-CT, bone marrow, EBER)
Transplantation	Haploidentical allo-HSCT	FBA conditioning
	Day +11	Klebsiella pneumoniae sepsis
	Day +20	Neutrophil engraftment
	Day +32	Platelet engraftment
Follow-up	2 months	Chimerism 99.66%, EBV negative, CMV positive
	6 months	EBV undetectable, discontinuation of anti-rejection medications
	1 year	Completion of chidamide maintenance
	35 months	Sustained CR

Key diagnostic and therapeutic milestones are summarized, including initial presentation, referral, diagnosis of ANKL with HLH, etoposide plus dexamethasone, P-Gemox induction, complete remission, haploidentical allo-HSCT, engraftment, post-transplant complications, chidamide maintenance, and durable remission at 35 months. HLH, hemophagocytic lymphohistiocytosis; ANKL, aggressive NK-cell leukemia; NGS, next-generation sequencing; CR, complete remission; allo-HSCT, allogeneic hematopoietic stem cell transplantation; FBA, fludarabine + busulfan + cytarabine; EBV, Epstein–Barr virus; CMV, cytomegalovirus.

## Discussion

The diagnosis of ANKL complicated by HLH poses a significant challenge, particularly for non-hematology specialists. HLH can be triggered by infections (especially EBV) or malignancies, making it essential to exclude underlying cancer (14). ANKL is frequently associated with HLH and carries a dismal prognosis (15). Our patient’s favorable outcome was attributable to timely diagnosis. Therefore, ANKL should be a leading differential in patient presenting with HLH, especially when EBV viremia and rapid deterioration are present.

A definitive diagnosis of ANKL requires a multimodal approach. While bone marrow aspirate may reveal large granular lymphocytes, it lacks specificity (16). Flow cytometry is the cornerstone, sensitively detecting aberrant NK cells even at low levels (17). The typical immunophenotype is CD2^+^, sCD3^−^, cCD3^+^, CD56^+^, CD16^−^, CD57^−^, with cytotoxic granule expression (18). In our case, flow cytometry identified 2.06% abnormal NK cells, demonstrating its sensitivity. The absence of surface CD3 and CD57 with strong CD56 expression distinguishes ANKL from T-LGLL (sCD3^+^, CD57^+^) (2). Immunohistochemistry confirmed CD3ϵ, Granzyme B, and EBER positivity. Biopsy of enlarged lymph nodes, spleen, liver, or skin lesions is also diagnostically valuable when indicated.

Genomic studies have elucidated ANKL’s molecular landscape. Our patient’s NGS revealed mutations in JAK1, ASXL1, KMT2D, and FAS, consistent with the known mutational profile. Huang et al. identified JAK/STAT pathway mutations in 48% of ANKL patients, with additional mutations in TP53 (34%), TET2 (28%), CREBBP (21%), and MLL2 (21%) (19). JAK/STAT activation has been reported to drive MYC expression and metabolic reprogramming in ANKL models (20); whether this pathway was functionally relevant in our patient remains undetermined. Dufva et al. reported mutations in epigenetic modifiers (50%), DDX3X (29%), STAT3 (21%), and RAS-MAPK genes (21%) (21). The JAK1 Ser703Ile alteration has been reported as a gain-of-function variant in other contexts. In this case, paired germline testing excluded a germline origin, confirming it as a somatic mutation, and serial variant allele frequency analysis showed that this mutation became undetectable after treatment, supporting its potential role in disease biology. Similarly, ASXL1 and KMT2D are epigenetic regulators frequently mutated in ANKL (22), and FAS mutations may contribute to immune evasion and apoptosis resistance (23), but these interpretations remain speculative in the present case. Accordingly, the molecular findings are presented as descriptive and hypothesis-generating rather than mechanistic. Notably, EBV-negative ANKL is associated with older age and higher TP53 mutation frequency (82% vs. 25%) (24, 25). Our EBV-positive patient with JAK1 and epigenetic mutations fits the typical profile.

The therapeutic approach to ANKL has evolved significantly. Anthracycline-based regimens like CHOP are largely ineffective due to P-glycoprotein-mediated multidrug resistance (26). L-asparaginase has been a breakthrough, exploiting NK cells’ asparagine synthetase deficiency. P-Gemox (pegaspargase, gemcitabine, oxaliplatin) has shown efficacy in advanced NK/T-cell lymphoma, with an ORR of 80.9% and a CR rate of 52.4% in a published series (10), and it is generally considered to have a more favorable toxicity profile than SMILE (27). In our patient, CR was achieved after two cycles of P-Gemox; however, because he also received etoposide plus dexamethasone for HLH control, subsequent allo-HSCT, and one year of chidamide maintenance, the durable remission cannot be attributed to P-Gemox alone. The contribution of P-Gemox should therefore be interpreted within the context of this sequential multimodal strategy. SMILE showed superior efficacy (2-year OS 30.1% vs. 7.0%) but with significant toxicity (12). AspaMetDex (L-asparaginase + methotrexate + dexamethasone) offers a better toxicity profile (28), and VIDL and DDGP are other effective options (29, 30).

Allo-HSCT is the only curative modality for ANKL. Patients receiving allo-HSCT had a 2-year OS of 37.2% vs. 3.5% without (12), with 5-year OS ~27.3% (11). A meta-analysis confirmed a pooled HR of 0.47 (31). Remission prior to transplant is critical (12); event-free patients at 12 months post-transplant have a 5-year OS of 85.2% (12). Our patient achieved CR, underwent haploidentical HSCT, and has maintained remission for 35 months with full chimerism.

Emerging therapies include PD-1 inhibitors, which have shown activity in relapsed/refractory NK/T-cell lymphomas (32, 33), and may be particularly valuable given EBV upregulates PD-L1 (34). Ruxolitinib combined with P-Gemox achieved an ORR of 83.3% (35). Other agents under investigation include BCL-2 inhibitors, HDAC inhibitors, brentuximab vedotin, and daratumumab (36–38). The transferrin receptor 1 axis is also a promising target (39).

CNS relapse has been reported in ANKL, and standardized prevention and treatment approaches warrant further study (40, 41). Our patient received prophylactic intrathecal chemotherapy and showed no CNS involvement throughout follow-up. However, CNS prophylaxis should be individualized and its role prospectively evaluated in larger cohorts.

This report has several limitations. First, as a single case, the findings cannot establish causality or generalizability, and the favorable outcome may reflect the combined effects of multiple interventions rather than any single agent. Second, the molecular analyses showed that no functional validation was performed, and post-transplant variant clearance may be influenced by high donor chimerism. Third, safety information was collected retrospectively and may be incomplete. Fourth, the recommendation regarding CNS prophylaxis is not supported by this single case, and prospective studies are needed. Finally, follow-up, although extending beyond 35 months, remains limited for drawing definitive conclusions about long-term cure.

In conclusion, this case demonstrates that ANKL presenting with severe, life-threatening HLH and multiorgan dysfunction can be successfully managed with a multimodal approach. P-Gemox was effective in inducing remission in this patient with critical illness, although its specific contribution cannot be isolated from the other components of therapy. Haploidentical allo-HSCT, with its accessibility and curative potential, represents a viable option for patients who lack fully matched donors. The molecular characterization of ANKL through NGS provides insights into disease biology and potential therapeutic targets. With the growing armamentarium of targeted therapies including JAK inhibitors, PD-1/PD-L1 inhibitors, and anti-CD38 antibodies, the future holds promise for improved outcomes in this devastating disease. Early diagnosis, prompt initiation of L-asparaginase-based therapy, and timely referral for allo-HSCT may represent the cornerstones of successful management.

## Patient perspective

The patient reported that the initial presentation with persistent high-grade fever, skin petechiae, and progressive fatigue was frightening and confusing, as his symptoms had been initially attributed to viral myocarditis and other conditions at a local hospital. He expressed deep gratitude for the timely referral to the hematology department and the prompt multidisciplinary evaluation that led to an accurate diagnosis of ANKL with HLH. Regarding his treatment course, he described the P-Gemox chemotherapy as tolerable. He noted that the medical team clearly explained each step of the treatment plan, which helped alleviate his anxiety. The haploidentical transplant from his brother was physically demanding but emotionally meaningful, as his sibling’s donation gave him hope and strength. The period before chemotherapy, along with the pre-neutrophil engraftment period, was the most difficult experience, but he credited the intensive care and continuous support from the medical staff for his recovery. He reported that he now feels well and has returned to his normal daily activities, and he expressed profound appreciation to the entire clinical team and his family for their unwavering support throughout his 35-month journey.

## Data Availability

The raw data supporting the conclusions of this article will be made available by the authors, without undue reservation.
